# Perceptions of Planetary Health and Sustainability Among Canadian Radiation Oncology Professionals: A National Survey

**DOI:** 10.7759/cureus.116941

**Published:** 2026-09-25

**Authors:** Stephanie Quon, Philip Wong, Zahra Kassam, Natalie Rozanec, Kristopher Dennis, Adam Gabara, Conrad Bayley, Daria Comsa, Joanne Alfieri, David Palma, Isabelle Vallieres, Shilo Lefresne

**Affiliations:** 1 Faculty of Medicine, University of British Columbia, Vancouver, CAN; 2 Temerty Faculty of Medicine, University of Toronto, Toronto, CAN; 3 Princess Margaret Cancer Centre, University Health Network, Toronto, CAN; 4 Stronach Regional Cancer Centre, Southlake Health, Newmarket, CAN; 5 Department of Radiation Oncology, University of Toronto, Toronto, CAN; 6 Division of Radiation Oncology, University of Ottawa, Ottawa, CAN; 7 Division of Radiation Oncology, McMaster University, Hamilton, CAN; 8 Division of Radiation Oncology, University of Calgary, Calgary, CAN; 9 Radiation Medicine Program, Stronach Regional Cancer Centre, Southlake Health, Newmarket, CAN; 10 Division of Radiation Oncology, McGill University Health Centre, Montreal, CAN; 11 Department of Radiation Oncology, London Health Sciences Centre, London, CAN; 12 Department of Radiotherapy, BC Cancer-Victoria, Victoria, CAN; 13 Department of Radiotherapy, BC Cancer-Vancouver, Vancouver, CAN

**Keywords:** climate change, healthcare emissions, planetary health, radiation oncology, sustainability

## Abstract

Background: Climate change poses increasing risks to human health and healthcare delivery, including increased cancer burden, treatment disruptions, and exacerbation of comorbidities. Despite growing international attention to sustainable cancer care, Canadian data on professional perceptions, institutional practices, and priorities for action within radiation oncology remain limited.

Objective: This study aimed to assess perceptions, knowledge, and workplace practices related to planetary health and sustainability among Canadian radiation oncology professionals.

Methods: A cross-sectional electronic survey was distributed to Canadian radiation oncology professionals and trainees through the Canadian Association of Radiation Oncology (CARO) membership email list and departmental distribution across cancer centers. The 27-item questionnaire was developed based on previously validated climate perception and sustainability surveys, including SASSY and CHANT, and assessed perceptions of climate change and health impacts, workplace sustainability practices, and priorities for action. Responses were anonymous and analyzed using descriptive statistics.

Results: A total of 108 respondents completed the survey, including radiation oncologists, therapists, physicists, nurses, and trainees from nine provinces. Climate change was rated as a personal priority by 73% (n = 79/108) of respondents, and 75% (n = 81/108) agreed that it would increase cancer incidence. Support for prioritizing low-carbon, low-waste cancer care was high (77%; n = 83/108). However, only 34% (n = 37/108) reported the presence of a sustainability initiative at their cancer center, and 62% (n = 67/108) felt sustainability was not a workplace priority. Strong support was expressed for CARO-led initiatives, including member education on planetary health (77%; n = 83/108), sustainability recommendations for clinical practice (76%; n = 82/108), and evaluation of radiation oncology’s carbon footprint (69%; n = 75/108). Interest in reducing CARO ASM emissions was high, with 58% (n = 63/108) supporting at least a 30% reduction.

Conclusion: While Canadian radiation oncology professionals recognize the relevance of climate change to cancer care and support action in sustainability, institutional implementation remains limited. Future work should evaluate strategies to engage broader professional groups and increase awareness and participation in sustainability initiatives in radiation oncology.

## Introduction

Climate change is an urgent public health crisis with far-reaching consequences for Canadians. It is associated with increased frequency and severity of extreme weather events, deteriorating air quality, threatened water and food security, and changing patterns of infectious disease [1,2]. In Canada, climate-related health risks are intensifying and are projected to impose substantial health and economic burdens, leading to an estimated $246 billion in annual societal costs and over 270,000 hospitalizations and premature deaths per decade [3]. These impacts disproportionately affect vulnerable populations and exacerbate existing health inequities [4,5].

The healthcare system itself is both affected by and contributes to climate change. Disruptions to supply chains, infrastructure, and staffing during climate disasters threaten continuity of care, especially for patients with chronic diseases such as cancer [6-8]. At the same time, the Canadian healthcare sector emits 33 million tons of CO₂ equivalent (tCO₂e) annually, representing 4.6% of national emissions, which is among the highest per capita globally [9]. Hospitals, pharmaceuticals, and physician services are key contributors [3].

Radiation oncology contributes to healthcare-related carbon emissions in multiple ways, including the electricity demand of linear accelerators used for external beam radiation therapy (EBRT), repeated patient travel for multi-fraction treatment courses, and energy use within radiation facilities [10,11]. Life cycle analyses estimate that a 25-fraction EBRT course in the United States produces over 4.3 tCO₂e, with approximately 74% attributable to building energy use and 26% to transportation [10]. During the COVID-19 pandemic, shifts toward hypofractionation and increased use of virtual care reduced departmental carbon footprints by 39% without increasing adverse outcomes [12]. These findings suggest that changes in treatment delivery models may reduce the environmental impact of radiation oncology while maintaining quality of care.

Despite the growing recognition of climate threats, the integration of sustainability into oncology practice remains limited. Surveys, largely drawing respondents from the United Kingdom and the United States, show high awareness among oncology professionals globally: nearly 99% acknowledge healthcare’s role in climate change, and 88% want further education [13]. National bodies such as the Canadian Medical Association advocate for net-zero emissions by 2050 and call for education, low-carbon practices, and Indigenous-led adaptation strategies [14-16]. In radiation oncology specifically, the American Society for Radiation Oncology has incorporated resource stewardship through its Choosing Wisely recommendations, which discourage low-value radiation treatments and promote evidence-based care that can also reduce unnecessary resource use and associated emissions [17]. Compared with professional societies in North America, the European Society for Radiotherapy and Oncology has taken earlier and more structured steps toward integrating environmental sustainability into its strategic framework, including initiatives focused on sustainable radiotherapy practice and environmental impact reduction [18]. However, barriers such as limited institutional support, time, and training persist globally [19-21].

To advance national action, the Canadian Association of Radiation Oncology (CARO) created a Climate Action and Sustainability Working Group in 2023. As little is known about Canadian radiation oncology professionals’ knowledge, attitudes, and practices regarding planetary health, this study aims to address that gap by surveying a national sample of radiation oncology professionals. Understanding these perspectives is essential for designing effective educational strategies and institutional efforts toward more sustainable cancer care.

## Materials and methods

Study design and participants

The study followed the Checklist for Reporting Results of Internet E-Surveys (CHERRIES) guidelines [22]. The protocol was reviewed and approved by the University of British Columbia BC Cancer Research Ethics Board (H25-00136). The literature was reviewed to identify previously validated surveys assessing climate change perceptions among the general public and healthcare professionals, as well as prior publications examining oncology-specific perspectives on climate change.

The study utilized a cross-sectional survey design to assess perceptions, knowledge, and priorities related to climate action and sustainability among Canadian radiation oncology professionals. Eligible participants included healthcare professionals (e.g., radiation oncologists, radiation therapists, medical physicists, nurses) and trainees affiliated with a Canadian cancer center or who were members of CARO.

The survey was distributed electronically. All CARO members received an invitation via email through the CARO Secretariat. To broaden reach and include non-members, the principal investigator directly contacted radiation therapy department heads across Canadian cancer centers, requesting dissemination to departmental staff. As the survey was additionally disseminated by department heads to departmental staff, the total number of individuals who ultimately received the survey could not be determined; therefore, a definitive response rate could not be calculated. The survey link was not advertised through public websites, social media, online forums, or banner ads, and therefore, there was no web-based context that could bias respondent selection. Participation was entirely voluntary and anonymous. By completing the survey, participants provided implied consent. Prior to beginning the survey, participants were informed of the study's purpose, estimated completion time, eligibility criteria, and the anonymity of their responses. No incentives to complete the survey were offered.

Survey instrument

The survey instrument was developed by the CARO Climate Action and Sustainability Working Group and administered via the Checkbox survey platform. Established instruments, including the Yale Climate Change Communication surveys, the Climate, Health, and Nursing Tool (CHANT), and the Sustainability Attitudes and Support Survey (SASSY), informed the development of survey items. The resulting adapted survey instrument underwent internal pretesting but was not subsequently formally validated. The survey consisted of 27 questions organized into four main sections. The “Demographics” section captured information such as gender identity, province or territory of practice, professional role, years in practice, and primary practice setting. Regions were grouped into Western (British Columbia, Alberta, Manitoba, Saskatchewan), Central (Ontario, Quebec), and Atlantic (Nova Scotia, New Brunswick, Newfoundland and Labrador). The “Basic Perceptions and Knowledge” section assessed the personal importance of climate change, self-reported knowledge across various climate-related topics, and prior exposure to climate-focused education through formats such as lectures, journal articles, and webinars. The “Center Assessment” section evaluated participants’ perceptions of the extent to which their institutions prioritize sustainability, implement emissions-reducing strategies, and support sustainable clinical practices. Finally, the “Priorities for CARO” section gauged participants’ awareness of the CARO Climate Action and Sustainability Working Group and collected feedback on preferred roles, actions, and educational strategies for CARO to advance sustainability initiatives. The full list of survey questions is available in Appendix A.

The survey included multiple-choice questions, Likert-scale items, and open-text responses. It addressed topics such as the perceived impacts of climate change on cancer care, institutional efforts in sustainability, and support for specific climate-related initiatives and policies. The survey was designed to take approximately 15 minutes to complete. The questionnaire underwent internal pretesting to assess usability, readability, and clarity. Conditional branching and adaptive questioning were applied where appropriate to reduce respondent burden. Respondents were able to review and modify their answers before final submission using back buttons. No randomization of item order was used. The survey was delivered over four pages/screens. The platform was optimized for both desktop and mobile use.

Data collection and integrity

Data was collected over a seven-week period between April 16, 2025, and June 6, 2025. To preserve data quality, several mechanisms were used to minimize duplicate or invalid responses. The survey platform recorded anonymized session data and timestamps without collecting IP addresses or using cookies. No registration or login was required. Responses completed in under one minute were flagged as implausible and excluded. View, participation, and completion rates were not calculated due to the anonymous design and lack of user-identifying tracking mechanisms. No automatic completeness or consistency checks were applied, and all questions were optional, with a non-response option available. All data were stored on encrypted servers accessible only to the research team.

Statistical analysis

Categorical variables were summarized using frequencies and percentages. Post hoc exploratory subgroup analyses were conducted to examine whether responses differed according to respondent role, years in practice, region of practice, and prior planetary health education. Categorical responses were compared using chi-squared (χ²) tests. For selected exploratory comparisons involving Likert-scale responses, mean scores were compared between groups using independent-samples t-tests. Statistical significance was defined as a two-sided p-value < 0.05. Given the exploratory nature of these analyses and the small sample size, no adjustment for multiple comparisons was performed. Additionally, no statistical weighting or other corrections were applied, as the sample was not intended to be nationally representative. Statistical analysis and the creation of figures were conducted in R version 4.1.3 (R Foundation for Statistical Computing, Vienna, Austria, https://www.R-project.org/) [23].

## Results

Respondent demographics

The survey invitation was distributed via email to approximately 631 CARO members, although the number of additional individuals who may have received the survey through secondary distribution channels could not be quantified. A total of 142 responses were received, of which 108 were complete, corresponding to a completion rate of 76% (n = 108/142). Partial responses were excluded from the analysis. The summary of respondent demographics is provided in Table 1.

**Table 1:** Demographic and practice characteristics of survey respondents

Category	Count	Percentage
Total responses	108	
Gender identity		
Woman	54	50%
Man	49	45%
Other	1	1%
Prefer not to respond	4	4%
Years in practice		
Trainee	13	12%
<5 years	16	15%
5-10 years	18	17%
11-15 years	17	16%
>15 years	44	41%
CARO member status		
Members	40	37%
Non-members	31	29%
Role		
Radiation oncologist	79	74%
Radiation therapist	10	9%
Medical physicist	9	8%
General practitioner in oncology	1	1%
Other	8	7%
Practice setting		
Academic center	83	77%
Community hospital	21	19%
Community clinic	6	6%
Province of practice		
British Columbia	45	42%
Ontario	26	24%
Alberta	10	9%
Nova Scotia	10	9%
Quebec	10	9%
Manitoba	3	3%
New Brunswick	1	1%
Newfoundland and Labrador	1	1%
Saskatchewan	1	1%
Prior training received on planetary health		
Podcasts	53	49%
Journal articles	50	46%
Session at a medical conference	46	43%
Rounds at a local or regional center	40	37%
Webinars	27	25%
Lecture in medical school or undergraduate training	19	18%
Dedicated courses or certifications	6	6%
Lecture in residency or postgraduate training	5	5%
Conference on climate change and/or planetary health	5	5%
Other	18	17%

Perceptions of climate change importance and impact on cancer care

When asked about the importance of climate change personally, 41% (n = 44/108) rated it as “5” (extremely important) on a 5-point Likert scale, and 32% (n = 35/108) rated it as “4” (very important). Self-reported knowledge of climate change varied, with 32% (n = 35/108) feeling “very informed” (rating of 4 out of 4), 53% (n = 57/108) moderately informed (rating of 3 out of 4), and only 1% (n = 1/108) reporting “not informed” (rating of 1 out of 4).

Most respondents agreed that climate change would likely affect access to healthcare in Canada (67%; n = 72/108 agree or strongly agree), and 75% (n = 81/108) agreed or strongly agreed that climate change would increase cancer incidence and exacerbate comorbidities. Similarly, respondents agreed that oncologists should understand planetary health (74%; n = 80/108 agreed or strongly agreed). Support for incorporating planetary health into medical education was notable, with 63% (n = 68/108) agreeing or strongly agreeing. Interest in reducing the ecological impact of cancer care was high, with 77% (n = 83/108) agreeing or strongly agreeing that low-carbon, low-waste practices should be prioritized. Lastly, 71% (n = 77/108) agreed or strongly agreed that they are interested in learning more about reducing the ecological impact of their practice. A full breakdown of these ratings is presented in Figure 1.

**Figure 1:**
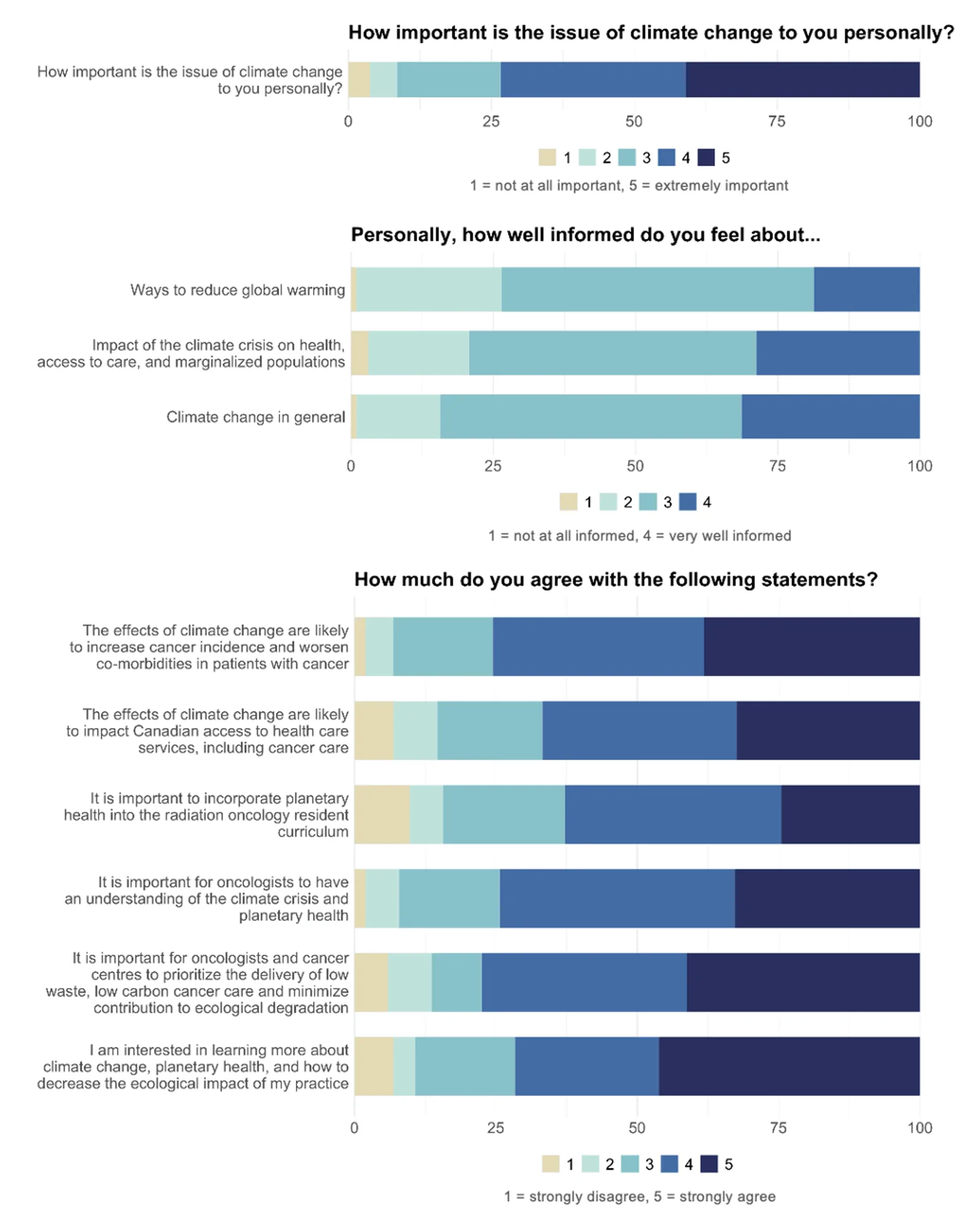
Distribution of survey responses regarding the importance of climate change, and agreement with statements about its health and cancer care impacts Respondents rated the items using 4- or 5-point Likert scales. Bars represent the percentage of respondents selecting each rating for each question.

Post hoc exploratory analysis by role, years in practice, region, and educational exposure identified several patterns. Trainees were the most likely to rate climate change as personally important, with 92% (n = 12/13) selecting 4 or 5 on a 5-point importance scale. In contrast, only 64% (n = 28/44) of respondents with over 15 years in practice rated it as 4 or 5. Mean personal importance scores were higher among trainees than among respondents with over 15 years in practice (4.23 ± 0.60 vs 3.95 ± 1.19), although this difference was not statistically significant (p = 0.27). When asked whether climate change is likely to impact access to cancer care in Canada, rated on a 5-point agreement scale, 85% (n = 11/13) of trainees selected 4 or 5, compared to just 47% (n = 8/17) of those with 11-15 years in practice. Mean agreement scores for this item were higher among trainees than among respondents with 11-15 years in practice (4.08 ± 0.95 vs 3.21 ± 1.37), although this difference did not reach statistical significance (p = 0.07).

Agreement with the statement that climate change is likely to affect access to cancer care varied modestly across regions. Mean agreement scores on a 5-point Likert scale were 3.70 ± 1.19 in Western Canada, 3.85 ± 1.15 in Central Canada, and 4.17 ± 1.03 in Atlantic Canada. Exploratory pairwise t-tests did not identify statistically significant differences between Western and Central Canada (p = 0.55), Western and Atlantic Canada (p = 0.18), or Central and Atlantic Canada (p = 0.38). In British Columbia specifically, 86% (n = 39/45) rated the importance of climate change as 4 or 5 out of 5, compared to 73% (n = 46/63) across the rest of Canada. Mean personal importance scores were similar between respondents in British Columbia and those in the rest of Canada (4.09 ± 0.90 vs 3.97 ± 1.18, p = 0.55). Respondents who had received prior education on climate or planetary health were slightly more likely to rate climate change as highly important (49%; n = 25/51 rated it as 5 out of 5) than those who had not (35%; n = 20/57). Mean personal importance scores were also slightly higher among respondents with prior planetary health education than among those without prior education (4.04 ± 1.06 vs 3.60 ± 1.14), although this difference was not statistically significant (p = 0.44).

Workplace sustainability practices

The majority (62%; n = 67/108) of respondents felt that sustainability and environmental stewardship were not a priority for their cancer center, and 55% (n = 59/108) believed their workplace is not doing enough to reduce emissions and waste. Only 34% (n = 37/108) reported the presence of a sustainability program at their center, and 50% (n = 54/108) were unsure. Common workplace sustainability practices included recycling (72%; n = 78/108), limiting waste (36%; n = 39/108), composting (30%; n = 32/108), and supporting active transportation for commuting (30%; n = 32/108). However, only 5% (n = 5/108) reported initiatives promoting plant-based diets, and only 1% (n = 1/108) reported efforts to end financial relationships with fossil fuel-dependent industries. An overview of all reported sustainability efforts is provided in Figure 2.

**Figure 2:**
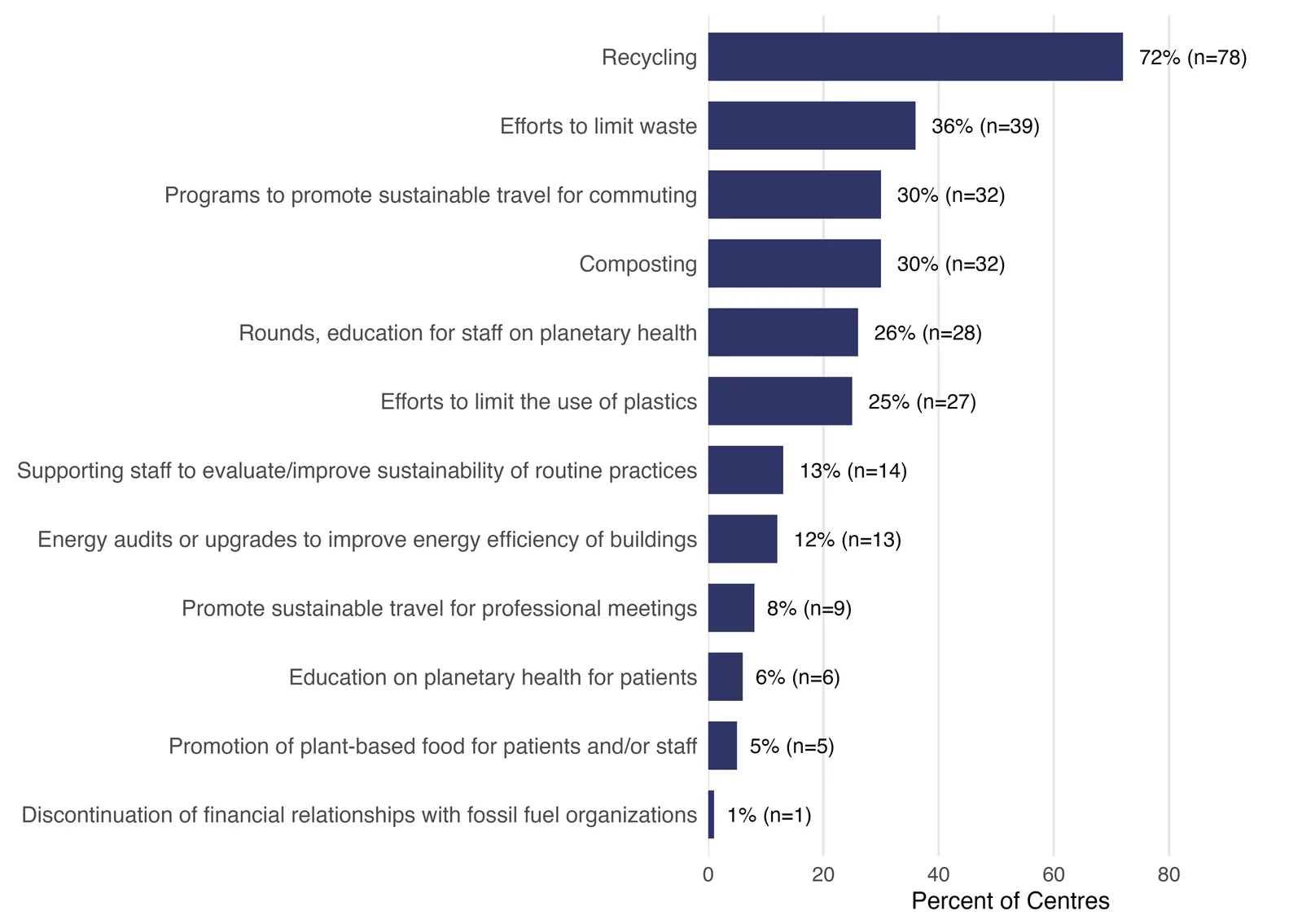
Reported workplace sustainability practices across cancer centers

Exploratory provincial-level breakdowns suggested regional differences in sustainability engagement. For example, 51% (n = 23/45) of respondents from British Columbia believed sustainability was a priority at their cancer center, compared to 42% (n = 11/26) in Ontario and 0% (n = 0/10) in Alberta, though this difference did not reach statistical significance (p = 0.13, χ² = 4.08, df = 2, Cramer’s V = 0.22). Sustainability programs were reported by 37% (n = 22/59) of respondents in Western Canada and 36% (n = 13/36) in Central Canada, while Atlantic Canada had the lowest reported presence, with only 1 respondent out of 12 (8%) identifying an initiative. This difference in the reported presence of sustainability programs was not statistically significant (p = 0.14, χ² = 3.89, df = 2, Cramer’s V = 0.19).

CARO’s role and organizational leadership

There was strong support among respondents for CARO to take a leadership role in climate action. The two most supported actions were providing education to CARO members about climate change and planetary health (77%; n = 83/108) and providing recommendations for low-waste, low-carbon, sustainable cancer care (76%; n = 82/108). Respondents also endorsed a variety of strategies to improve the sustainability of CARO’s meetings. Top suggestions included implementing a zero-waste policy for swag and consumables (76%; n = 82/108) and encouraging active and public transportation for travel (70%; n = 76/108). There was less support for initiatives such as carbon offsetting for travel (43%; n = 46/108) and fully virtual meetings (11%; n = 12/108). An overview of the full responses is provided in Figure 3.

**Figure 3:**
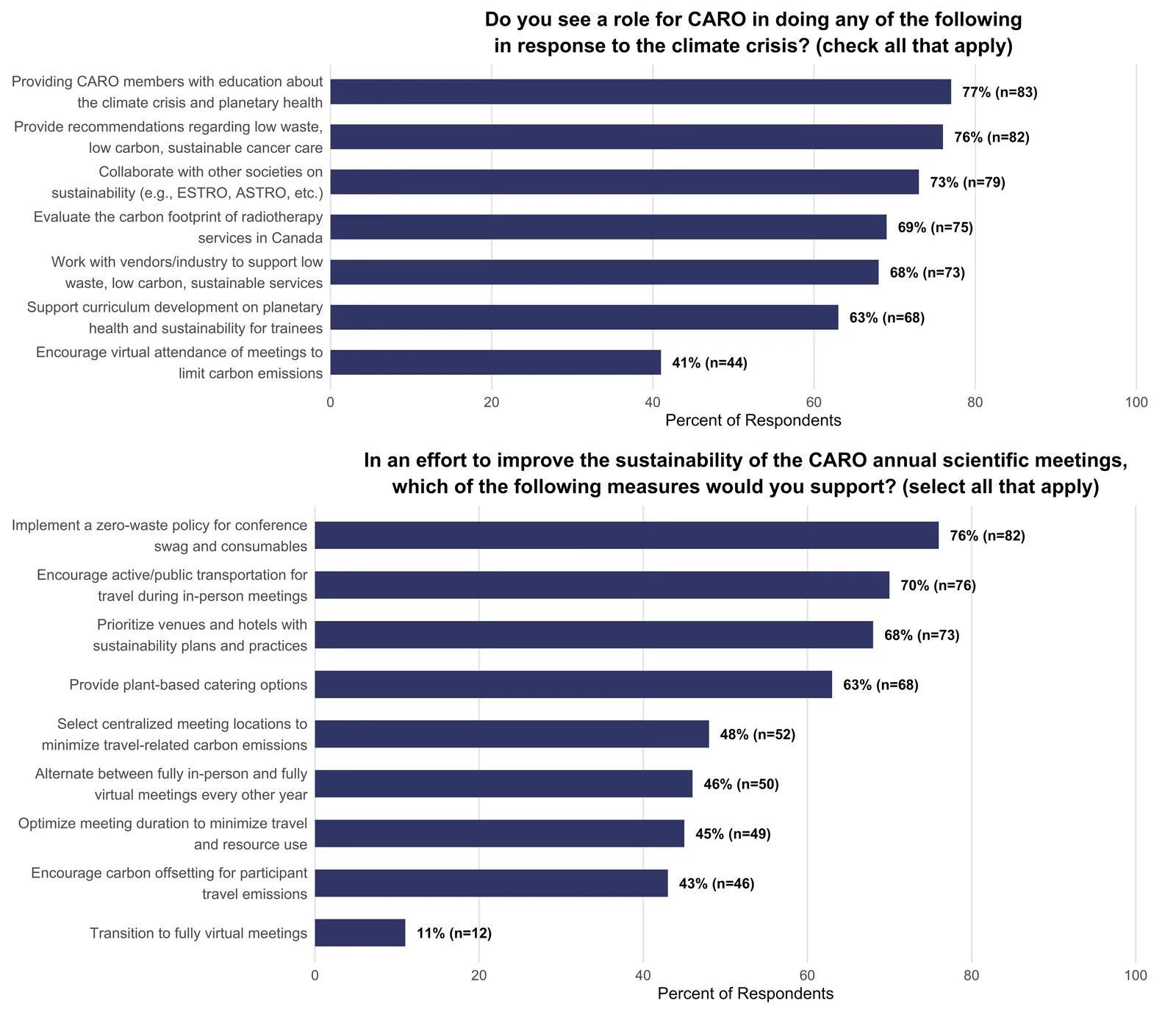
Respondent perspectives on CARO’s role in climate action and sustainability strategies for annual scientific meetings

When asked about reducing carbon emissions associated with CARO’s annual scientific meetings, most respondents supported action. Nearly a third (27%; n = 29/108) supported a 30% emissions reduction, followed by 23% (n = 25/108) supporting a 50% reduction, and 18% (n = 19/108) supporting a 10% reduction. Nineteen percent (n = 20/108) believed no reduction was necessary, while 8% (n = 9/108) favored the most ambitious option of a 70% reduction.

Regarding educational preferences, the majority of respondents were interested in sessions at the CARO Annual Scientific Meeting (55%; n = 59/108) or webinars (49%; n = 53/108) as formats for learning more about sustainability and planetary health. Other preferred modalities included the CARO website (45%; n = 49/108), mailing lists (30%; n = 32/108), and structured educational modules (24%; n = 26/108).

Exploratory analyses suggested potential differences in support for sustainability strategies by province and years of practice. Respondents from Ontario (85%; n = 22/26) and Quebec (90%; n = 9/10) were among the most likely to support a zero-waste conference policy (p = 0.074, χ² = 14.31, df = 8, Cramer’s V = 0.37), while British Columbia respondents strongly supported active and public transportation (76%; n = 34/45) (p = 0.14, χ² = 12.26, df = 8, Cramer’s V = 0.34). Support for plant-based catering was highest in Ontario (81%; n = 21/26), and lowest in provinces such as Manitoba (0%; n = 0/3) and Alberta (30%; n = 3/10) (p = 0.02, χ² = 18.17, df = 8, Cramer’s V = 0.41).

Differences by years of practice were also observed. Those with 5-10 years in practice were most likely to support a zero-waste policy (94%; n = 15/16) (p < 0.001, χ² = 18.47, df = 4, Cramer’s V = 0.41), while trainees and physicians with <5 years were more likely to support carbon offsetting (46%; n = 6/13 and 50%; n = 5/10, respectively) (p = 0.52, χ² = 3.23, df = 4, Cramer’s V = 0.17) and centralized meeting locations (62%; n = 8/13 and 38%; n = 4/10, respectively) (p = 0.36, χ² = 4.36, df = 4, Cramer’s V = 0.20).

Respondents who had prior education on planetary health were more likely to endorse CARO taking action on climate-related issues, though these differences were not statistically significant. For example, 84% (n = 43/51) of respondents with prior planetary health education supported CARO providing education on planetary health, compared to 69% (n = 39/57) of respondents without prior exposure (p = 0.22, χ² = 1.50, df = 1, Cramer’s V = 0.12). Similarly, 72% (n = 37/51) of respondents with prior education supported the development of a planetary health curriculum for resident physicians, compared to 54% (n = 31/57) of those without prior education (p = 0.30, χ² = 1.07, df = 1, Cramer’s V = 0.10).

## Discussion

This national survey suggests that Canadian radiation oncology professionals are both concerned about climate change and its implications for cancer care, and supportive of sustainability initiatives and institutional leadership in education and policy. Respondents also reported limited institutional prioritization of sustainability, suggesting a gap between individual concern and system-level action. To our knowledge, this is the first Canadian survey examining climate-related perceptions and sustainability priorities within radiation oncology, providing an important profession-specific perspective within an evolving health policy landscape.

The high levels of concern about climate change, perceived relevance to patient care, and reported gaps in institutional action observed in this survey are consistent with findings from prior surveys of healthcare professionals internationally. In a multinational survey of 4,654 health professionals across 12 countries, the majority agreed that climate change poses a growing threat to human health, yet many reported limited ability to act due to time constraints, insufficient resources, and lack of institutional support [24]. In an international survey of oncology professionals, nearly all respondents recognized climate change and healthcare’s contribution to emissions, and 88% expressed interest in further education, though only about half reported workplace preparedness for climate-related events [13]. Across academic and hospital-based surveys, clinicians consistently report that climate change education is relevant to clinical practice, but feel insufficiently trained and unsupported to act [25-29]. Altogether, these findings suggest that the attitudes reported by Canadian radiation oncology professionals may align with perspectives reported by the broader healthcare workforce.

Respondents’ concerns about the impacts of climate change on cancer care are supported by the literature, which shows that climate change affects not only cancer incidence but also the delivery of timely and equitable care. Climate-related events disrupt care by damaging infrastructure, interrupting power and supply chains, constraining transportation, and exacerbating workforce shortages [2,7,30]. Extreme weather events have been linked to cancelled or delayed oncology appointments, interruptions in radiotherapy schedules, medication shortages, and temporary or prolonged closures of treatment facilities resulting in adverse outcomes [2,7,30]. Radiation oncology is particularly vulnerable to extreme weather due to its reliance on uninterrupted power, specialized equipment, and time-sensitive treatment schedules [2,30]. These challenges affect both low- and high-income settings and have prompted calls for cancer centers to strengthen emergency preparedness, infrastructure resilience, and environmental sustainability to maintain care during climate-related disruptions [11,31]. These risks are especially salient in Canada, where climate-related disasters are increasing in frequency and severity, and where geographic distance already complicates equitable access to oncology services [32].

Despite high levels of concern, respondents describe substantial barriers to institutional action in sustainability, consistent with prior literature citing limited leadership, insufficient funding, and competing clinical priorities [33-36]. These challenges were reflected in our findings, where most respondents felt sustainability was not prioritized within their institutions. Despite these barriers, there are clear examples of health systems that have successfully integrated sustainability into practice. The United Kingdom’s National Health Service has embedded sustainability into system governance through a net-zero strategy, mandatory emissions reporting, and dedicated leadership structures, while maintaining high standards of care [37-40]. In Canada, initiatives such as the Canadian Coalition for Green Health Care, CASCADES, and Choosing Wisely demonstrate how sustainability can be aligned with quality, appropriateness, and value-based care, emphasizing waste reduction and resource stewardship rather than framing environmental action as a competing healthcare system priority [41]. The CARO Climate Action and Sustainability Working Group has also developed a radiation oncology-specific sustainability implementation toolkit to support institutional action and guide environmentally responsible practices in radiotherapy services [42].

The strong support for CARO-led climate action observed in this survey directly reflects CARO’s stated value of optimizing the sustainability of cancer care in Canada while minimizing its effects on the health of the planet, and is consistent with broader Canadian medical priorities articulated by the Canadian Medical Association and the Royal College of Physicians and Surgeons of Canada [43,44]. At the system level, national healthcare oversight bodies are also beginning to formally incorporate sustainability into healthcare governance. Health Standards Organization and Accreditation Canada have announced commitments to addressing the impacts of climate change within healthcare systems, and climate-related indicators are expected to be incorporated into future accreditation standards for healthcare organizations, which may drive system-wide institutional accountability for sustainability practices [45].

Respondents in this survey also strongly supported sustainability education despite reporting limited prior exposure during training, mirroring national data showing uneven integration of planetary health across Canadian medical education and continuing professional development [27]. While education alone is insufficient to drive practice change, it is a critical foundation for engagement, leadership, and institutional accountability. Practical models include competency-based curricula, continuing professional development modules, and the integration of sustainability principles into existing education initiatives [28,46]. Efforts to address this gap are underway nationally, including initiatives led by the Association of Faculties of Medicine of Canada and ongoing consideration of planetary health competencies in future updates to the CanMEDS framework and Royal College accreditation standards, reflecting growing recognition that physicians require training to address climate-related health system challenges [27,47-49]. These approaches align with national calls to equip clinicians with the skills needed to respond to climate-related health system risks.

Exploratory analyses performed suggested regional variation in perceptions of institutional sustainability efforts. Respondents from British Columbia were more likely to report the presence of sustainability initiatives, whereas respondents from Prairie and Atlantic provinces more often perceived sustainability as a low organizational priority. However, respondents were disproportionately from British Columbia (42%; n = 45/108), exceeding the province’s share of Canadian radiation oncologists relative to larger provinces such as Ontario. This overrepresentation may reflect greater engagement with sustainability initiatives or other structural factors, such as British Columbia’s radiation oncology workforce being centralized under a single provincial cancer agency, which may facilitate more coordinated survey participation compared with provinces operating across multiple health authorities. Overall, these findings should be interpreted cautiously, as the analyses were conducted post hoc and limited by small regional sample sizes. Rather than indicating definitive national patterns, these differences are best viewed as hypothesis-generating findings that may reflect variation in health authority leadership or policy environments. The results may inform targeted regional strategies, leveraging existing strengths in some jurisdictions while addressing gaps in others, and may be of value to organizations such as CARO, the Canadian Medical Association, and the Royal College of Physicians and Surgeons of Canada in guiding future research and policy development.

This study has several important limitations. Although the survey was informed by established instruments and underwent internal pretesting, the adapted instrument was not formally validated following modification. Due to the method of survey distribution, the total number of individuals who received the survey could not be determined, precluding calculation of a definitive response rate and limiting assessment of potential non-response bias. While national in scope, the survey sample represents a relatively small proportion of the Canadian radiation oncology workforce, which may limit the generalizability of the findings. The sample also had an uneven distribution of professional roles and geographic regions, with radiation oncologists comprising the majority of respondents and disproportionate representation from academic centers and British Columbia. The modest response volume may also reflect volunteer or self-selection bias, whereby respondents with greater interest in, or awareness of, planetary health and sustainability may have been more likely to participate. The exploratory post hoc analyses by region, role, and years in practice are limited by the small sample size and lack of adjustment for multiple comparisons, increasing the potential for type I error. These findings should be interpreted as hypothesis-generating and warrant further evaluation. Lastly, our survey relied on self-reported data, which may be subject to reporting bias. Assessment of institutional sustainability practices was based on respondents’ perceptions and was not independently verified; therefore, responses may not fully reflect practices at the institutional level. Despite these limitations, as a cross-sectional survey focused on respondents’ perceptions, attitudes, and priorities, these findings provide a useful baseline assessment of current perspectives and can help inform future research and initiatives. This study also has notable strengths, including its national reach, inclusion of multiple professional roles, and high response completeness. Future research may focus on recruiting larger and more representative samples, incorporating objective assessments of institutional practices, and evaluating the impact of educational and policy interventions over time to support climate-resilient oncology care in Canada.

## Conclusions

Canadian radiation oncology professionals who responded to this national survey consistently identified climate change as relevant to both their personal values and professional practice, and many anticipated downstream effects on cancer incidence, comorbidities, and access to care. Respondents expressed strong support for low-waste, low-carbon approaches to cancer care and for expanded education in planetary health. At the same time, respondents perceived limited institutional prioritization and reported relatively few center-level sustainability initiatives, suggesting a gap between professional interest and organizational implementation within the surveyed cohort. These findings highlight an opportunity to support clinicians through clearer institutional guidance and integration of sustainability within existing quality improvement, governance, and educational structures. Supporting sustainability efforts may help translate individual engagement into coordinated, system-level action that supports the long-term resilience and sustainability of cancer care in Canada.

## Appendices

Appendix A

Full Survey

Section 1: Demographics

1. Gender identity

·     Woman

·     Man

·     Non-binary/gender non-conforming

·     Other: _______

·     Prefer not to respond

2. Location of practice

·     British Columbia

·     Alberta

·     Saskatchewan

·     Manitoba

·     Ontario

·     Quebec

·     New Brunswick

·     Nova Scotia

·     Prince Edward Island

·     Newfoundland and Labrador

·     Yukon

·     Northwest Territories

·     Nunavut

·     Prefer not to respond

·     Other: _______

3. Professional role

·     Radiation oncologist

·     Medical physicist

·     Radiation therapist

·     General practitioner in oncology

·     Nurse practitioner

·     Other: _______

4. Years in practice

·     Trainee

·     <5 years

·     5-10 years

·     11-15 years

·     >15 years

5. Primary practice setting (select all that apply)

·     Academic center

·     Community hospital

·     Community clinic

·     Other: _______

Section 2: Basic perceptions and knowledge

6. How important is the issue of climate change to you personally?

·     1 - Not at all important

·     2

·     3

·     4

·     5 - Extremely important

7. How well-informed do you feel about the following? (Scale: 1 = not at all informed, 4 = very well-informed)

·     Climate change in general

·     The climate crisis and its impact on health, access to care, and marginalized populations

·     Ways to reduce global warming

8. What education or training have you received on climate-related topics? (Select all that apply)

·     Lecture in medical school or undergrad

·     Lecture in residency or postgrad

·     Local/regional rounds

·     Medical conference session

·     Dedicated conference on planetary health

·     Courses or certifications

·     Podcasts

·     Webinars

·     Journal articles

·     Other: _______

9. Level of agreement with the following statements (Scale: 1 = strongly disagree, 5 = strongly agree)

·     Climate change will impact access to cancer care

·     Climate change may increase cancer incidence or worsen comorbidities

·     Oncologists should understand the climate crisis

·     Planetary health should be in the radiation oncology curriculum

·     Low-waste, low-carbon care should be a priority

·     I am interested in learning more about sustainability in practice

Section 3: Center assessment

10. Is sustainability a priority at your cancer center?

·     Yes

·     No

11. Is your workplace doing enough to reduce emissions and waste?

·     Yes

·     No

·     Not sure

12. Is there a sustainability initiative at your center?

·     Yes

·     No

·     Not sure

13. Which sustainability practices does your center engage in? (Select all that apply)

·     Limiting plastic use

·     Waste reduction efforts

·     Composting

·     Recycling

·     Energy efficiency upgrades

·     Sustainable commuting

·     Sustainable travel for professional meetings

·     Plant-based food offerings

·     Staff education on planetary health

·     Patient education on planetary health

·     Support for sustainable clinical practices

·     Divestment from fossil fuel-heavy industries

·     Other: _______

Section 4: Priorities for CARO

14. Were you previously aware of CARO’s Climate Action and Sustainability Working Group?

·     Yes

·     No

15. What roles should CARO take in addressing climate change? (Select all that apply)

·     Member education

·     Trainee curriculum support

·     Evaluate radiotherapy’s carbon footprint

·     Provide sustainability recommendations

·     Work with vendors/industry

·     Collaborate with international organizations

·     Encourage virtual meetings

16. Target carbon emission reduction for CARO ASM (from in-person baseline)

·     0%

·     10%

·     30%

·     50%

·     70%

17. Which sustainability measures would you support for CARO meetings? (Select all that apply)

·     Fully virtual meetings

·     Alternate years: virtual vs. in-person

·     Centralized meeting locations

·     Encourage carbon offsetting

·     Optimize meeting duration

·     Choose sustainable venues/hotels

·     Zero-waste policies for swag and food

·     Plant-based catering options

·     Promote active/public local transportation

18. Preferred formats to learn more about planetary health (select all that apply)

·     Mailing list

·     Webinars

·     Sessions at CARO ASM

·     CARO website

·     Structured educational modules

·     Other: _______
